# Supplementary figures and images for: From paleness to albinism: Contribution of OCA2 exon 10 skipping to hypopigmentation
Source: PLoS Genet. 2025 Sep 25;21(9):e1011801. doi: 10.1371/journal.pgen.1011801 (PMC12463227; doi:10.1371/journal.pgen.1011801)

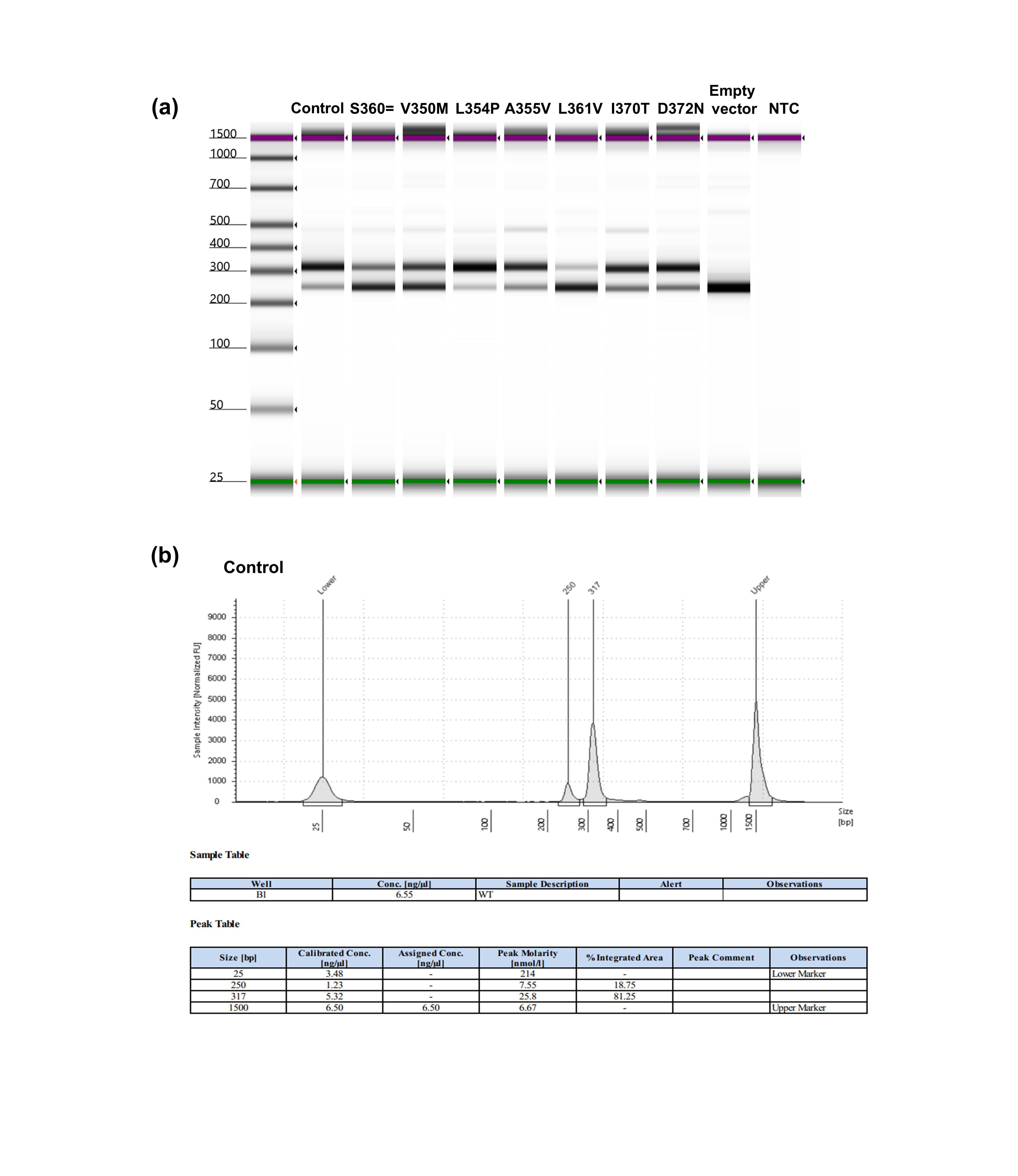

Supplement: S1 Fig — (a) Whole gel image of RT-PCR products corresponding to Fig 1b using D1000 DNA Screentape system on an Agilent TapeStation 4150 instrument. PCR products derived from minigene transcripts are detected through fluorescence around 300 bp. The upper (1500 bp) and lower (25 bp) markers, in D1000 loading dye, are size markers used as references for assessing the DNA fragments sizes in a sample. NTC = no-template control. (b) Electropherogram obtained following electrophoresis of RT-PCR products from the control minigene, shown in the gel above. The percentage integrated area (PIA) of the peak corresponding to the RT-PCR product without exon 10 is directly used to estimate the % of exon skipping as illustrated in the table below. (TIF) [file pgen.1011801.s001.tif]

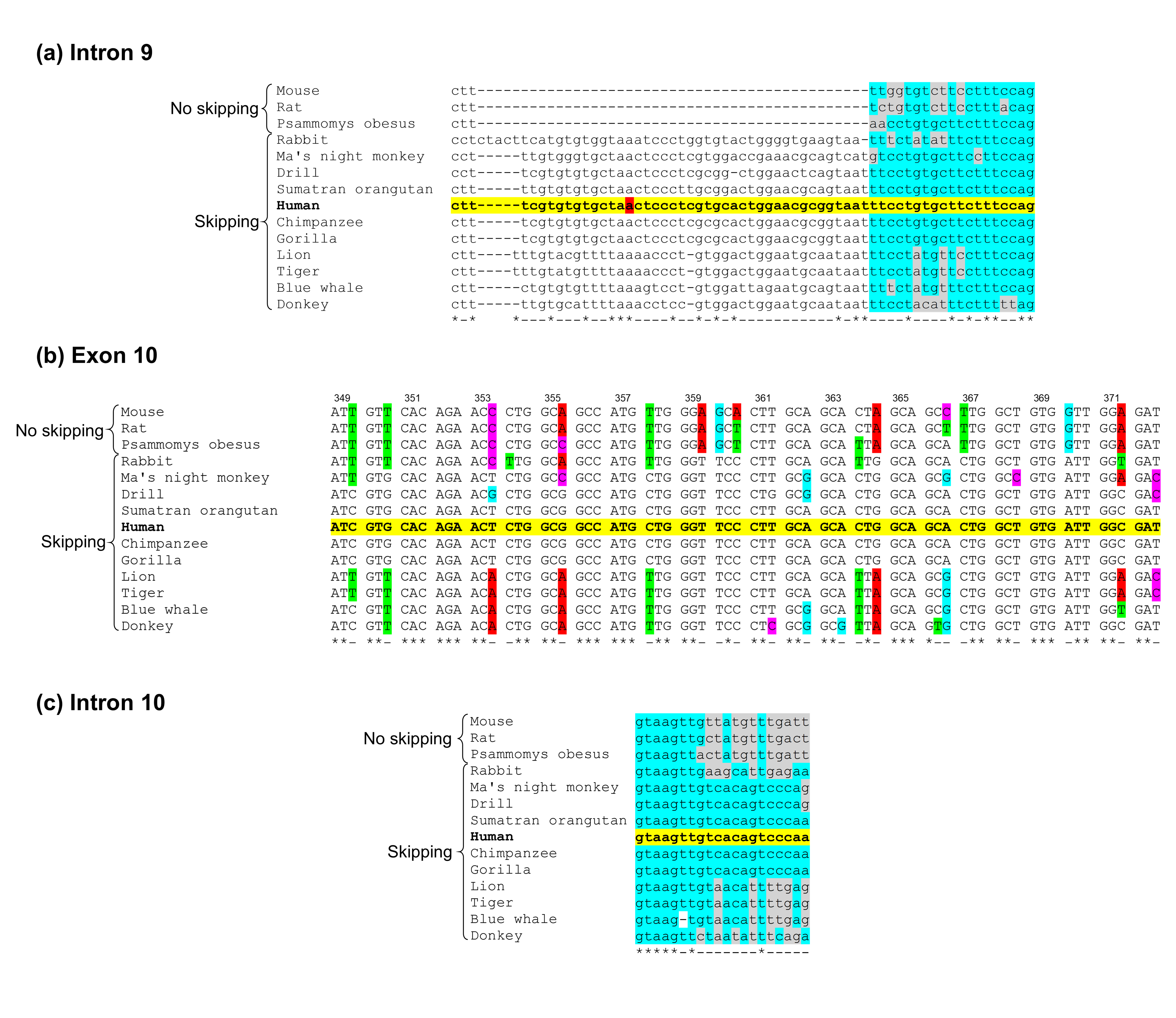

Supplement: S2 Fig — (a) Alignment of OCA2 intron 9 sequences. Sequences alignment was performed on Clustal Omega (https://www.ebi.ac.uk/jdispatcher/msa/clustalo). Conserved nucleotides are identified by * while non homologous nucleotides are indicated by -. The human sequence is hightlighted in yellow and the putative splicing branch point is in red, 47 bp upstream the intron 3′ splice site (note that for mouse, the branch point is predicted at -37 bp, not shown on the alignment). Intronic nucleotides near the exon highlighted in blue indicate highly conserved sequences across mammalian species. (b) Alignment of OCA2 exon 10 sequences. Codon positions are indicated above the alignment. Non conserved nucleotides are highlighted: green for T, pink for C, blue for G, red for A. (c) Alignment OCA2 intron 10, legend as for (a). (TIF) [file pgen.1011801.s002.tif]

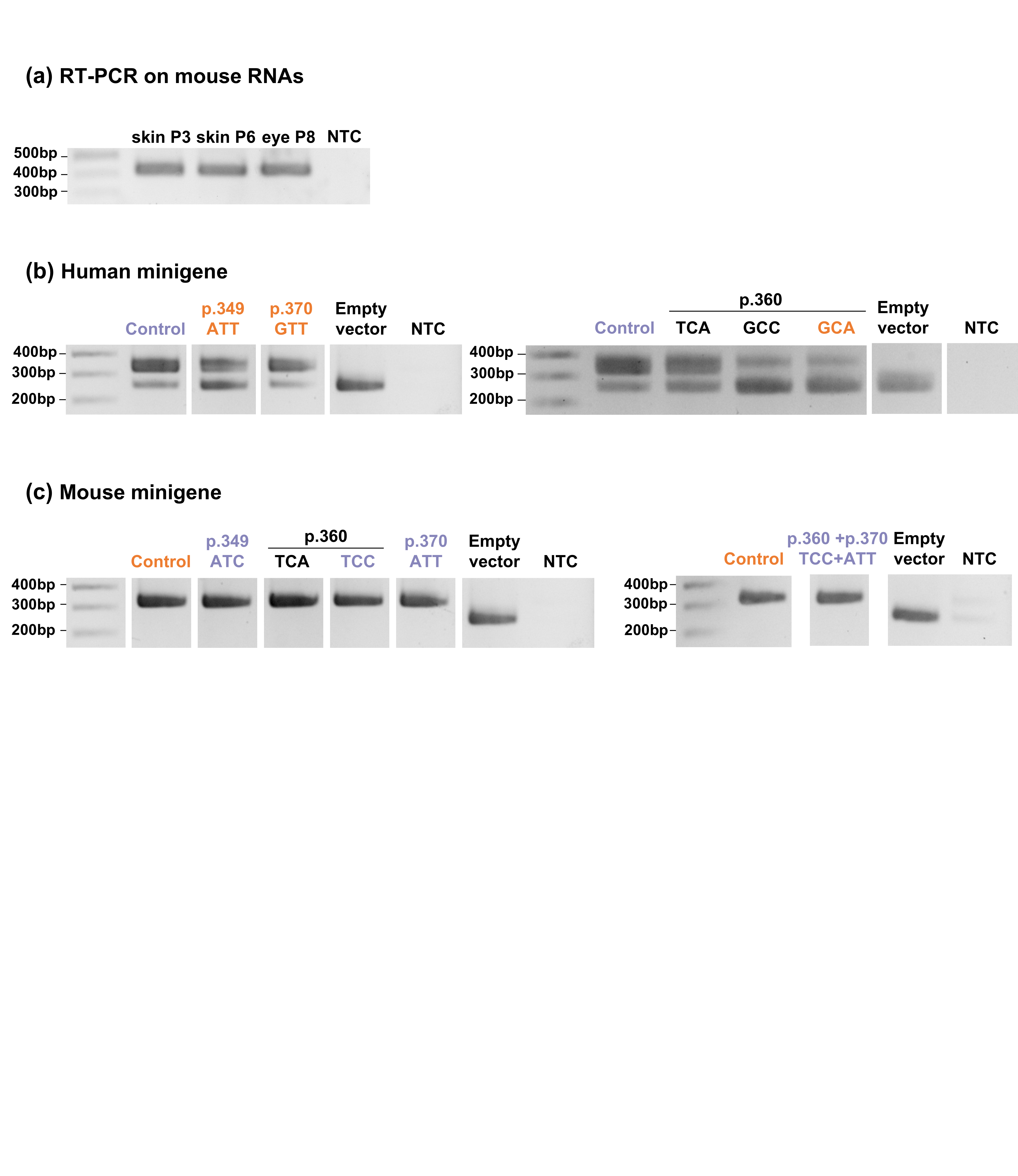

Supplement: S3 Fig — (a) Products of RT-PCR performed on mouse skin or optic cup RNAs using primers in exon 9 and 13 (see Methods) (b-c) Agarose gel electrophoresis of RT-PCR products of human (b) and mouse (c) minigene assay showing the effect of nucleotide swapping between human and mouse exon 10 sequences. Sequences of human origin are indicated in purple; sequences of murine origin, in red. NTC = no-template control. (TIF) [file pgen.1011801.s003.tif]

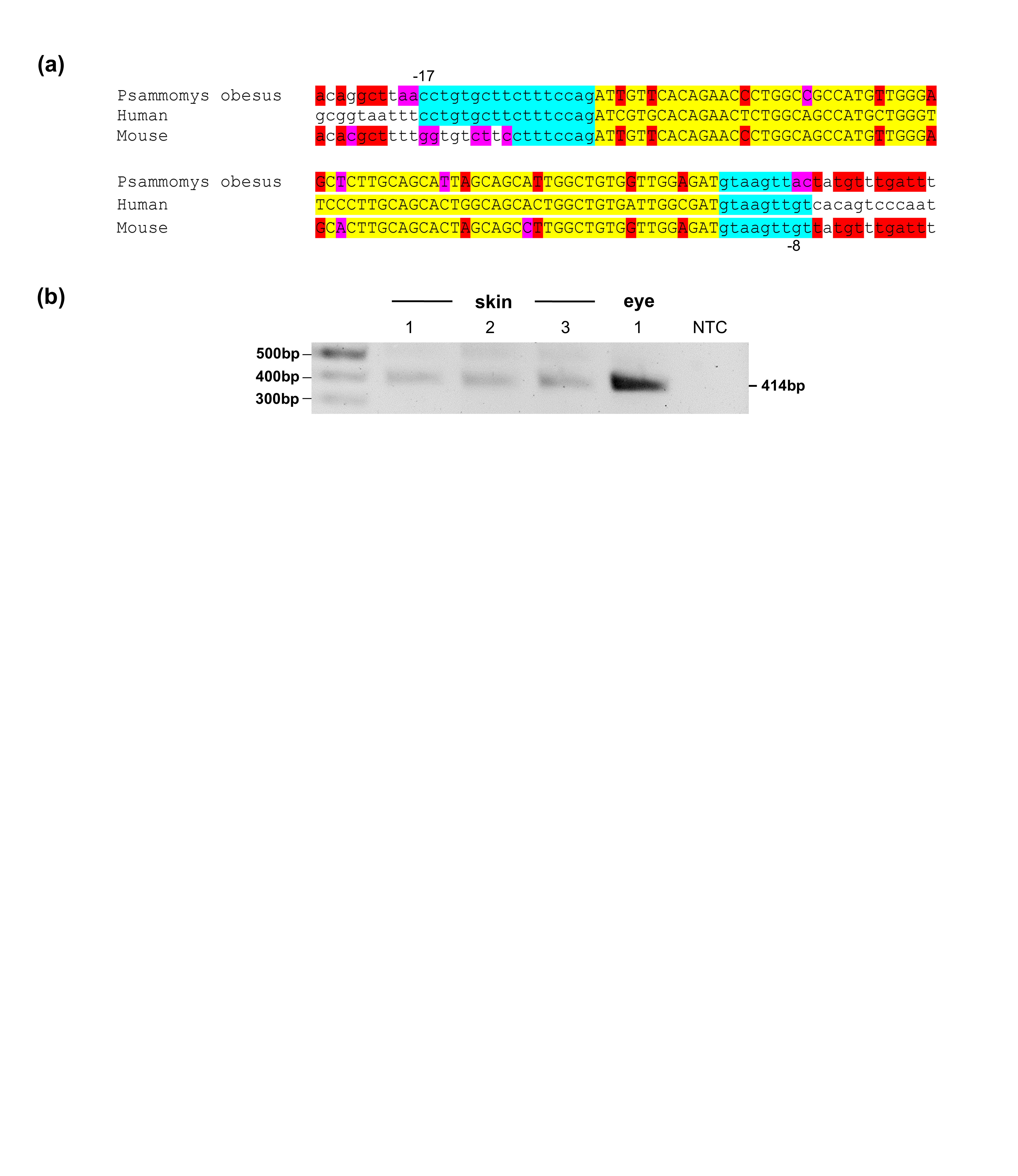

Supplement: S4 Fig — (a) Alignment between OCA2 exon 10 and surrounding sequences of human, mouse and Psammomys obesus (human: ENSG00000104044; mouse: ENSMUSG00000030450; Psammomys: NW 026590486.1 (1302928–1626514)). Sequence alignment was performed on Clustal Omega (https://www.ebi.ac.uk/jdispatcher/msa/clustalo). Upper case letters refer to exon sequence (also highlighted in yellow) and lower case letters to intronic sequences flanking the exon. Intronic nucleotides highlighted in blue indicate similar sequences between the species, red indicates homology between mouse and Psammomys but not human whereas pink indicates nucleotides that are specific to one of the 2 rodent species. (b), Agarose gel electrophoresis of RT-PCR products of Oca2 from Psammomys skin and eye total RNAs (primers in exon 8 and 12, see S1 Table). RT-PCR products correspond to the transcript correctly spliced with all exons (414 bp). This experiment was done on the skin of three different animals numbered 1 to 3, and on one eye of animal n°1. NTC = no-template control. (TIF) [file pgen.1011801.s004.tif]

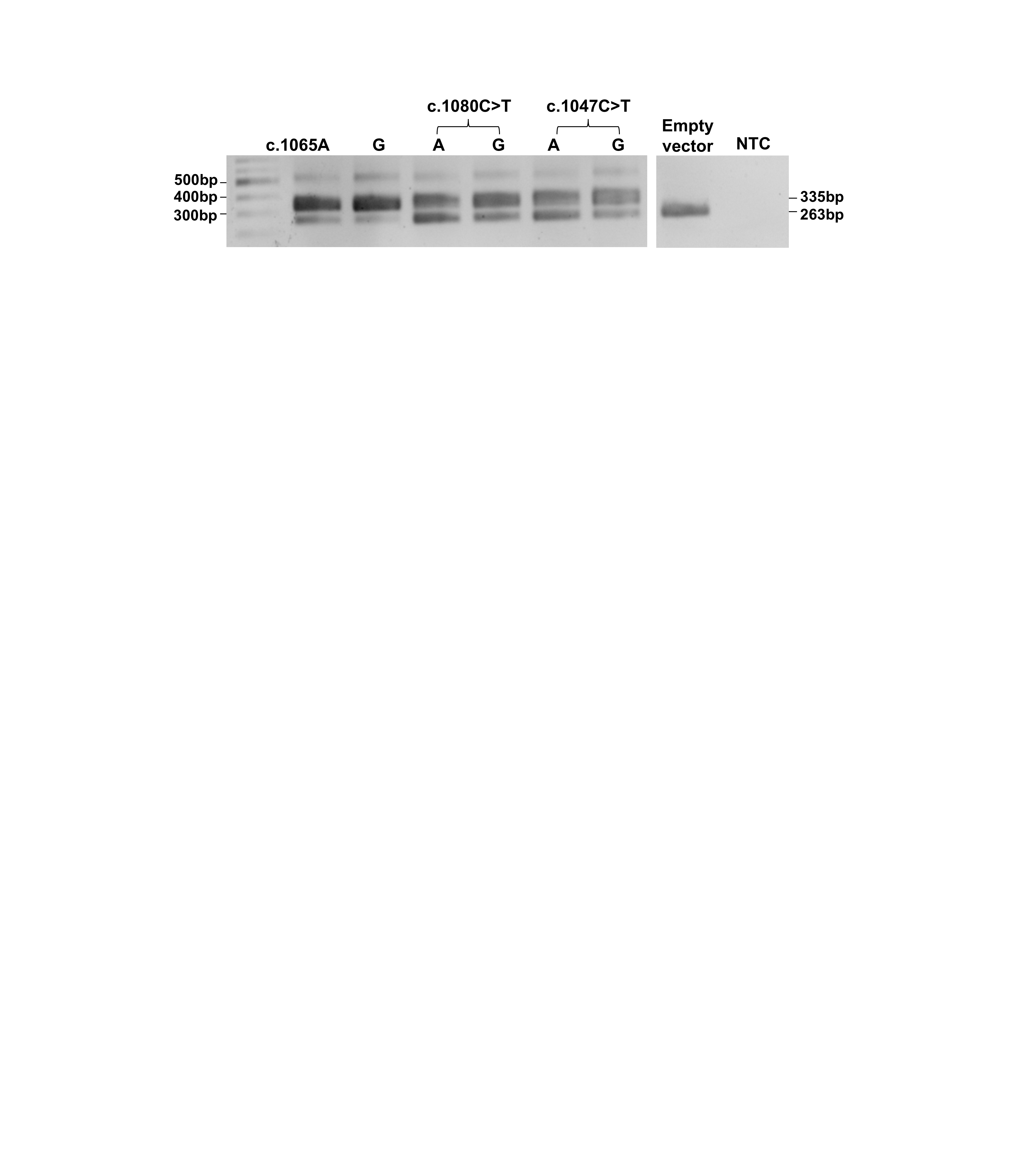

Supplement: S5 Fig — Minigene assay evaluated by conventional agarose electrophoresis showing the effect of c.1047C > T/p.Ile349 = equivalent to the increase in skipping induced by c.1080C > T that was precisely quantified by Screen tape analysis (Fig 5). Variant c.1047C > T is comparatively tested in cis to c.1065G or c.1065A as indicated. NTC: no-template control. (TIF) [file pgen.1011801.s005.tif]

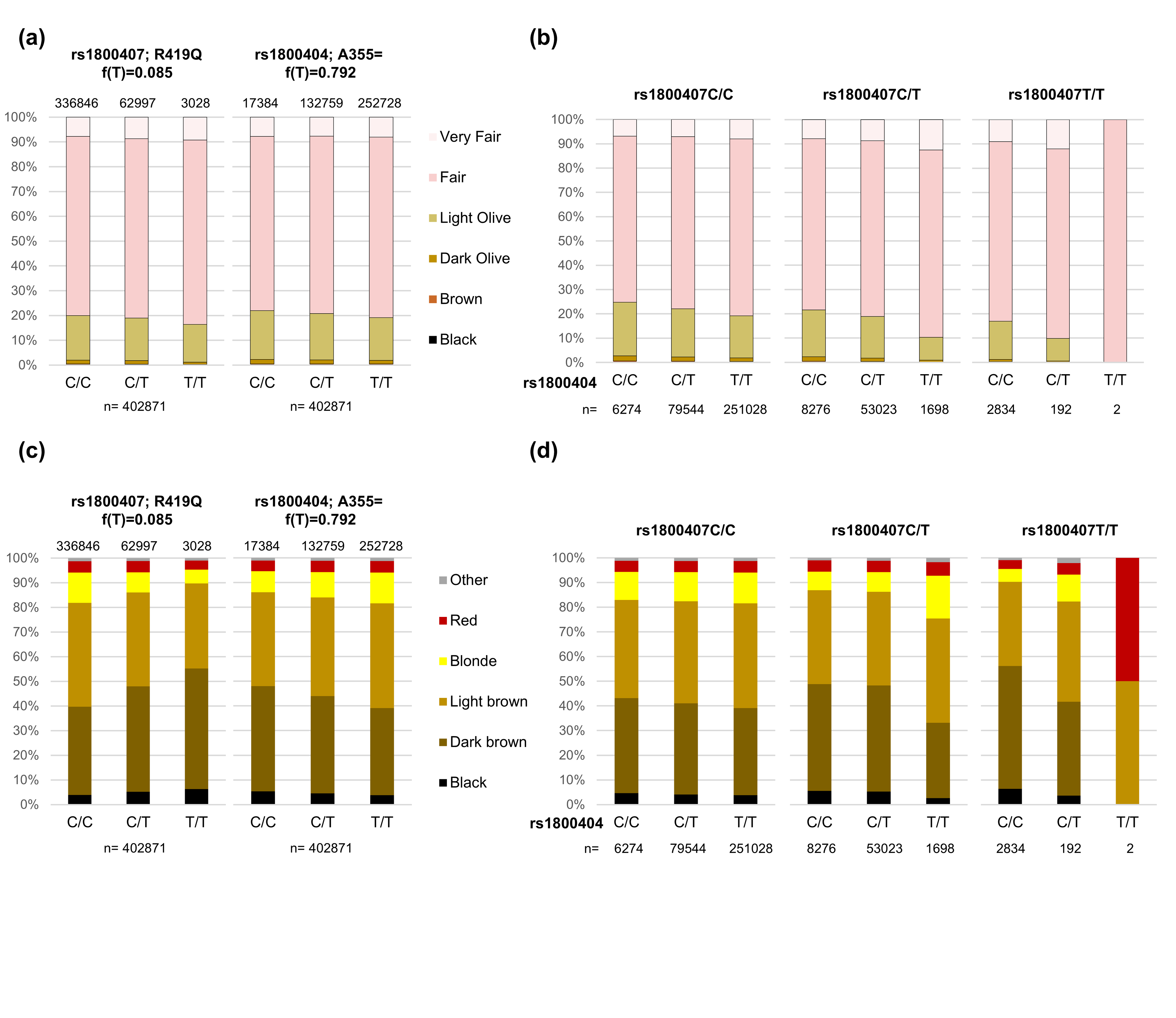

Supplement: S6 Fig — (a-b) Comparative association of rs1800407:C/T and rs1800404:C/T to skin colour. (c-d) Comparative association of rs1800407:C/T and rs1800404:C/T to hair colour. Association of skin colour (a) or hair colour (c) to the 3 genotypes (C/C, C/T, T/T) at rs1800407 (left) or rs1800404 (right) in the UK Biobank with European ancestry (n = 402871). Categories are indicated by corresponding colours. For each SNV analyzed separately, significant association is detected between the (T) allele and paleness of the skin (a). However, for the hair colour (c), the effect of rs1800407:C/T is opposite to what is expected, with (T) associated to darker rather than lighter hair colour. The effect of rs1800404-T, on the other hand, is in agreement with a contribution to paleness of both skin and hair (see text for statistical values). (b-d) Association of skin colour (b) or hair colour (d) to the 9 combinations of genotypes at rs1800404:C/T and rs1800407:C/T. Double heterozygotes with cis or trans segregation of the variants cannot be discriminated. This illustrates the strong association of rs1800404-T with hypopigmentation of the skin and hair independently of rs1800407-T as statistically shown in Fig 6. (TIF) [file pgen.1011801.s006.tif]

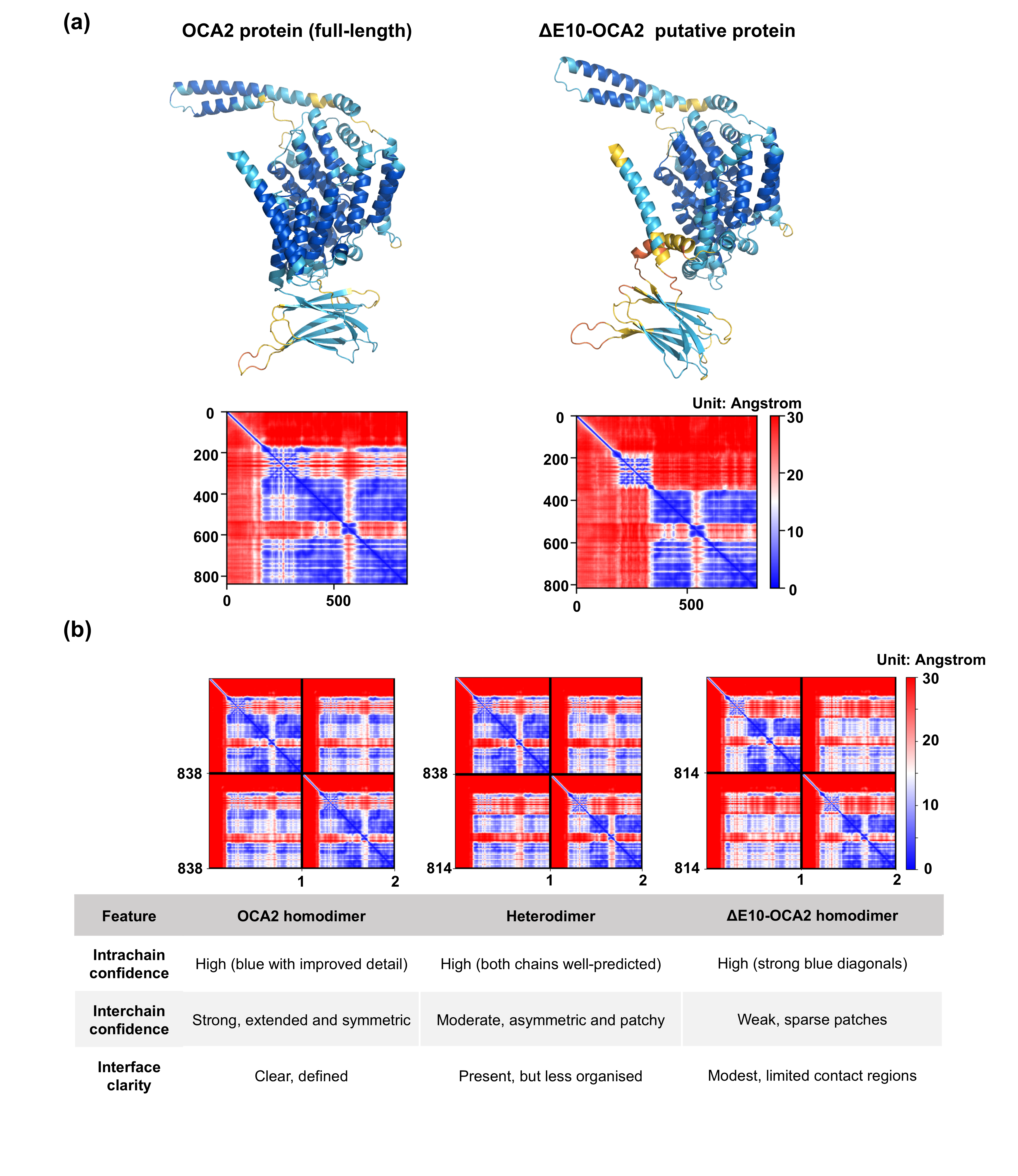

Supplement: S7 Fig — (a) Structural models of two ColabFold OCA2 monomers coloured by per-residue confidence (pLDDT). Models are truncated removing the first 165 residues representing the N-terminal disordered region. Models are coloured according to the pLDDT scale, where dark blue indicates high confidence (>90), light blue represents moderate confidence (70–90), yellow low confidence (50–70), and orange very low confidence (<50). Each model is shown in cartoon representation with both chains visible. Below: Predicted Aligned Error (PAE) maps the two monomer models. PAE maps visualise the predicted alignment error (in Angstroms) between residue pairs across and within chains. Blue regions indicate high confidence (low error), and red regions represent low confidence (high error), with values capped at 30 Å. Diagonal blocks correspond to intrachain predictions; off-diagonal blocks indicate interchain confidence. (b) PAE maps for three AlphaPulldown dimeric models. Same as above. Below: Comparative summary of PAE maps for the three dimer AlphaPulldown models. Each model is evaluated for intrachain confidence and interchain interaction quality based on PAE colour distribution. Intrachain confidence reflects the predicted accuracy of the individual monomer folds, while interchain confidence and interface clarity indicate the reliability and extent of the predicted dimer interface. (TIF) [file pgen.1011801.s007.tif]

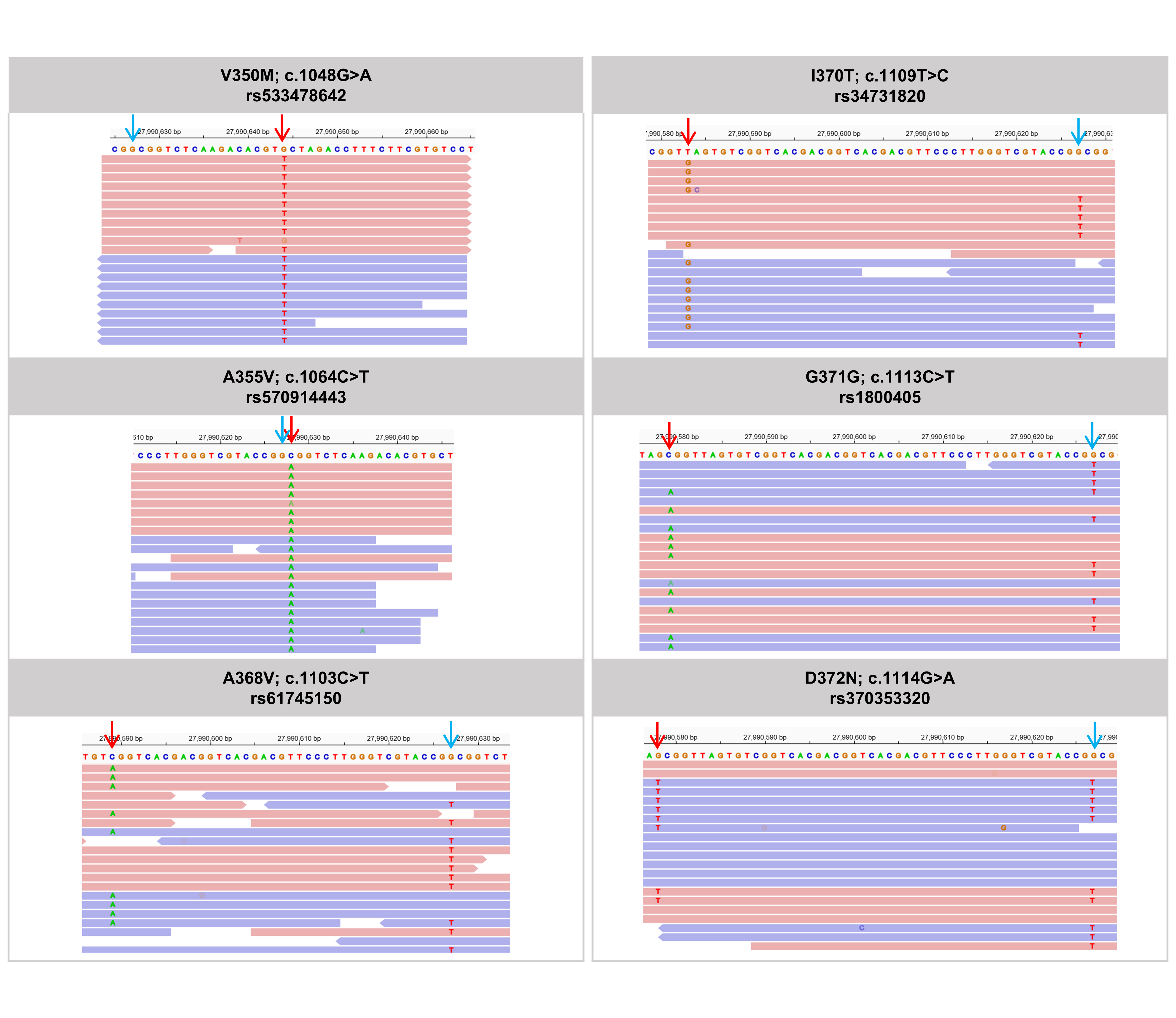

Supplement: S8 Fig — Sequencing data (GnomAd V4.0.1) from subjects carrying a rare missense or synonymous variant of exon 10 was examined for segregation with c.1065G > A. Number of homozygotes for each rare variant were: p.V350M (2 homozygotes); p.L354P (no homozygote); p.A355V (4 homozygotes); p.L361V (no homozygote); p.A368V (1 homozygote); p. I370T (9 homozygotes); p.G371= (479 homozygotes); p.D372N (1 homozygote). On all recovered sequences (n = 156), half were incomplete such that the haplotype could not be defined. On the remaining: all presumably healthy homozygotes carried the rare variant segregating in cis to c.1065(G), n = 6: 2 with p.V350M and 4 with p.A355V (see corresponding sequence panels). As for the heterozygous carriers, only 3 heterozygotes for the same variant (D372N) carried the variant in cis to c.1065G > A (last panel). Two were double heterozygotes and one was homozygote for c.1065G > A. All in all, no presumably healthy homozygote for a rare missense variant that enhances exon 10 skipping carried the variant in cis to c.1065G > A in agreement with the benign nature of such haplotype. The position of c.1065 is indicated by a blue arrow, the position of the rare variant by a red arrow. (TIF) [file pgen.1011801.s008.tif]
