# Supplementary material for: From paleness to albinism: Contribution of OCA2 exon 10 skipping to hypopigmentation
Source: PLoS Genet. 2025 Sep 25;21(9):e1011801. doi: 10.1371/journal.pgen.1011801 (PMC12463227; doi:10.1371/journal.pgen.1011801)
Supplement: S1 Table — (PDF) [file pgen.1011801.s009.pdf]

Human minigene 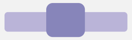		
Homologous recombination in pSPL3B vector		Forward : 5’ ACCAGAATTCTGGAGCTCGAGCAGTTGCCAAGGGAGCATA 3’
		Reverse : 5’ ATCCTGCAGCGGCCGCTCGAGCCAAACAGGACACCCTCATC 3’
Mutagenesis	c.1045-9T>G	Forward : 5’ GGTAATTTCCTGTGCTGCTTTCCAGATCGTGCAC 3’
		Reverse : 5’ GTGCACGATCTGGAAAGCAGCACAGGAAATTACC 3’
	c.1047C>T;p.Ile349=	Forward : 5’ TGTGCTTCTTTCCAGATTGTGCACAGAACTCTGGC 3’
		Reverse : 5’ GCCAGAGTTCTGTGCACAATCTGGAAAGAAGCACA 3’
	c.1048G>A ; p.Val350Met	Forward : 5’ GCTTCTTTCCAGATCATGCACAGAACTCTGG 3’
		Reverse : 5’ CCAGAGTTCTGTGCATGATCTGGAAAGAAGC 3’
	c.1061T>C ; p.Leu354Pro	Forward : 5’ GTGCACAGAACTCCGGCAGCCATGCTG 3’
		Reverse : 5’ CAGCATGGCTGCCGGAGTTCTGTGCAC 3’
	c.1064C>T ; p.Ala355Val	Forward : 5’ GCACAGAACTCTGGTAGCCATGCTGGGTTC 3’
		Reverse : 5’ GAACCCAGCATGGCTACCAGAGTTCTGTGC 3’
	c.1065A>G ; p.Ala355=	Forward : 5’ TGCACAGAACTCTGGCGGCCATGCTGGGTTC 3’
		Reverse : 5’ GAACCCAGCATGGCCGCCAGAGTTCTGTGCA 3’
	c.1078T>G ; p.Ser360Ala	Forward : 5’ GCCATGCTGGGTGCCCTTGCAGCAC 3’
		Reverse : 5’ GTGCTGCAAGGGCACCCAGCATGGC 3’
	c.1080C>A ; p.Ser360=	Forward : 5’ GCCATGCTGGGTTCACTTGCAGCACTGGC 3’
		Reverse : 5’ GCCAGTGCTGCAAGTGAACCCAGCATGGC 3’
	c.1078T>G + c.1080C>A	Forward : 5’ GCAGCCATGCTGGGTGCACTTGCAGCACTGGCA 3’
		Reverse : 5’ TGCCAGTGCTGCAAGTGCACCCAGCATGGCTGC 3’

	c.1081C>G ; p.Leu361Val	Forward : 5’ CATGCTGGGTTCCGTTGCAGCACTGG 3’
		Reverse : 5’ CCAGTGCTGCAACGGAACCCAGCATG 3’
	c.1108A>G ; p.Ile370Val	Forward : 5’ GCAGCACTGGCTGTGGTTGGCGATGTAAGTT 3’
		Reverse : 5’ AACTTACATCGCCAACCACAGCCAGTGCTGC 3’
	c.1109T>C ; p.Ile370Thr	Forward : 5’ CAGCACTGGCTGTGACTGGCGATGTAAGTTG 3’
		Reverse : 5’ CAACTTACATCGCCAGTCACAGCCAGTGCTG 3’
	c.1114G>A ; p.Asp372Asn	Forward : 5’ CACTGGCTGTGATTGGCAATGTAAGTTGTCACAG 3’
		Reverse : 5’ CTGTGACAACTTACATTGCCAATCACAGCCAGTG 3’
**Human minigene (intron 9: 73bp/ intron 10: 28bp)** 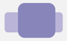		
Homologous recombination in pSPL3B vector		Forward : 5’ GGAGCTCGAGCGGCCGCGTCCACACAGGCTTTCGTGTG 3’
		Reverse : 5’ GATCCTGCAGCGGCCGCGCCAGGGATTGGGACTGTG 3’
**Human minigene (intron 9: 73bp/ intron 10: 85bp)** 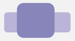		
Homologous recombination in pSPL3B vector		Same forward as intron 9 73bp
		Reverse : 5’ GATCCTGCAGCGGCCACCAGCGAAAGCCTGAATCC 3’
**Human minigene (intron 9: 73bp/ intron 10: 212bp)** 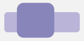		
Homologous recombination in pSPL3B vector		Same forward as intron 9 73bp
		Reverse : 5’ GATCCTGCAGCGGCCCCACTGGGATGTGAGTGTGTG 3’
**Human minigene (intron 9: 73bp/ intron 10: 330bp)** 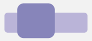		
Homologous recombination in pSPL3B vector		Same forward as intron 9 73bp
		Reverse : 5’ GATCCTGCAGCGGCCGCTAGGACGGTCCCCTCTAGTT 3’

**Human minigene (intron 9: 73bp/ intron 10: 429bp)**		
Homologous recombination in pSPL3B vector		Same forward as intron 9 73bp
		Reverse : 5’ ATCCTGCAGCGGCCGCTCGAGCCAAACAGGACACCCTCATC 3’
**Human minigene (intron 9: 326bp/ intron 10: 28bp)**		
Homologous recombination in pSPL3B vector		Forward : 5’ ACCAGAATTCTGGAGCTCGAGCAGTTGCCAAGGGAGCATA 3’
		Same reverse as intron 10 28bp
**Murine minigene**		
Homologous recombination in pSPL3B vector		Forward : 5’ GGAGCTCGAGCGGCCCTGAAGTCTCAATTAGATAAGGCAG 3’
		Reverse : 5’ GATCCTGCAGCGGCCTGGGTATCTCCAGGGCCTATG 3’
Mutagenesis	c.1045-9C>G	Forward : 5’ CACGCTTTTGGTGTCTTGCTTTCCAGATTGTTCAC 3’
		Reverse : 5’ GTGAACAATCTGGAAAGCAAGACACCAAAAGCGTG 3’
	c.1047T>C; p.Ile349=	Forward : 5’ GTCTTCCTTTCCAGATCGTTCACAGAACCCTGG 3’
		Reverse : 5’ CCAGGGTTCTGTGAACGATCTGGAAAGGAAGAC 3’
	c.1078G>T; p.Ala360Ser	Forward : 5’ GCAGCCATGTTGGGATCACTTGCAGCACTAG 3’
		Reverse : 5’ CTAGTGCTGCAAGTGATCCCAACATGGCTGC 3’
	c.1078G>T + c.1080A>C	Forward : 5’ GCAGCCATGTTGGGATCCCTTGCAGCACTAG 3’
		Reverse : 5’ CTAGTGCTGCAAGGGATCCCAACATGGCTGC 3’
	c.1108G>A; p.Val370Ile	Forward : 5’ GCAGCCTTGGCTGTGATTGGAGATGTAAGTT 3’
		Reverse : 5’ AACTTACATCTCCAATCACAGCCAAGGCTGC 3’

**Murine minigene (intron 9: 75bp/ intron 10: 20bp)** 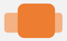		
Homologous recombination in pSPL3B vector		Forward : 5’ GGAGCTCGAGCGGCCGCCTTTTTCATTTGATACACATGAAGC 3’
		Reverse : 5’ GATCCTGCAGCGGCCGCAATCAAACATAACAACTTACATCTC 3’
**Minigene with mouse introns and human exon** 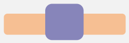		
Homologous recombination in pSPL3B vector		Murine minigene forward and reverse primers
Mutagenesis	c.1045-9C>G	Forward : 5’ CGCTTTTGGTGTCTTGCTTTCCAGATCGTGC 3’
		Reverse : 5’ GCACGATCTGGAAAGCAAGACACCAAAAGCG 3’
**Minigene with human introns and mouse exon** 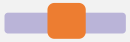		
Homologous recombination in pSPL3B vector		Human minigene forward and reverse primers
Mutagenesis	c.1045-9T>G	Forward : 5’ CGCGGTAATTTCCTGTGCTGCTTTCCAGATTGTTC 3’
		Reverse : 5’ GAACAATCTGGAAAGCAGCACAGGAAATTACCGCG 3’
**Minigene with human intron 9 and exon and mouse intron 10** 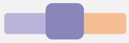		
Homologous recombination in pSPL3B vector		Forward : 5’ GGAGCTCGAGCGGCCTACTCCATCTGGCCTTCC 3’
		Murine minigene reverse primer
**Minigene with mouse intron 9 and human exon and intron 10** 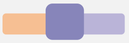		
Homologous recombination in pSPL3B vector		Murine minigene forward primer
		Human minigene reverse primer

Table S1: List of primers for homologous recombination in pSPL3B and mutagenesis. Human exon and introns sequences are shown in dark and light purple respectively. Mouse exon and introns are in dark and light orange.
