# Supplementary material for: From paleness to albinism: Contribution of OCA2 exon 10 skipping to hypopigmentation
Source: PLoS Genet. 2025 Sep 25;21(9):e1011801. doi: 10.1371/journal.pgen.1011801 (PMC12463227; doi:10.1371/journal.pgen.1011801)
Supplement: S2 Table — Informations were collected on Ensembl: http://www.ensembl.org/index.html except for Psammomys obesus informations were obtained on NCBI: https://www.ncbi.nlm.nih.gov/. Abbreviations: Δ10 = exon 10 skipped. (PDF) [file pgen.1011801.s010.pdf]

Species	Gene identification	Exon 10 or equivalent	Full length *OCA2* transcript<br>Δ10 *OCA2* transcript	
Mouse	ENSMUSG00000030450	No skipping	ENSMUST00000032633.12 Oca2-201	https://www.ensembl.org/Mus_musculus/Gene/Summary?db=core;g=ENSMUSG00000030450;r=7:55889508-56186266
Rat	ENSRNOG00000014465	No skipping	ENSRNOT00000108070.2 Oca2-202	https://www.ensembl.org/Rattus_norvegicus/Gene/Summary?db=core;g=ENSRNOG00000014465;r=1:116251796-116581838
Psammomys obesus	129676979	No skipping	XM_055607524.1	https://www.ncbi.nlm.nih.gov/gene/129676979
Rabbit	ENSOCUG00000003510	Skip	ENSOCUT00000003517.4 OCA2-201<br>ENSOCUT00000060813.1 OCA2-202	https://www.ensembl.org/Oryctolagus_cuniculus/Gene/Summary?db=core;g=ENSOCUG00000003510;r=17:78036010-78397189
Ma's night monkey	ENSANAG00000024732	Skip	ENSANAT00000031480.1 OCA2-201<br>ENSANAT00000031481.1 OCA2-202	https://www.ensembl.org/Aotus_nancymaae/Gene/Summary?db=core;g=ENSANAG00000024732;r=KZ202154.1:573255-828565
Drill	ENSMLEG00000033698	Skip	ENSMLET00000042501.1 OCA2-201<br>ENSMLET00000042507.1 OCA2-202	https://www.ensembl.org/Mandrillus_leucophaeus/Gene/Summary?db=core;g=ENSMLEG00000033698;r=KN974395.1:2623632-2969172
Sumatran ourangutan	ENSPPYG00000006278	Skip	ENSPPYT00000007412.3 OCA2-201<br>ENSPPYT00000036919.1 OCA2-202	https://www.ensembl.org/Pongo_abelii/Gene/Summary?db=core;g=ENSPPYG00000006278;r=15:2120028-2466892
Human	ENSG00000104044	Skip	ENST00000354638.8 OCA2-202<br>ENST00000353809.9 OCA2-201	https://www.ensembl.org/Homo_sapiens/Gene/Summary?db=core;g=ENSG00000104044;r=15:27754875-28099315
Chimpanzee	ENSPTRG00000006834	Skip	ENSPTRT00000079363.1 OCA2-201<br>ENSPTRT00000045837.5 OCA2-202	https://www.ensembl.org/Pan_troglodytes/Gene/Summary?db=core;g=ENSPTRG00000006834;r=15:8829677-9162797
Gorilla	ENSGGOG00000005900	Skip	ENSGGOT00000005933.3 OCA2-201<br>ENSGGOT00000030992.2 OCA2-202	https://www.ensembl.org/Gorilla_gorilla/Gene/Summary?db=core;g=ENSGGOG00000005900;r=15:6233317-6581604
Lion	ENSPLOG00000000481	Skip	ENSPLOT00000000764.1 OCA2-201<br>ENSPLOT00000000775.1 OCA2-202	https://www.ensembl.org/Panthera_leo/Gene/Summary?db=core;g=ENSPLOG00000000481;r=B4:26023193-26491686;t=ENSPLOT00000000764
Tiger	ENSPTIG00000005972	Skip	ENSPTIT00000006956.1 OCA2-201<br>ENSPTIT00000006959.1 OCA2-202	https://www.ensembl.org/Panthera_tigris_altaica/Gene/Summary?db=core;g=ENSPTIG00000005972;r=KE722366.1:800302-1104135
Blue whale	ENSBMSG00010005864	Skip	ENSBMST00010008870.1 OCA2-201<br>ENSBMST00010008877.1 OCA2-202	https://www.ensembl.org/Balaenoptera_musculus/Gene/Summary?db=core;g=ENSBMSG00010005864;r=7:2378692-2635994
Donkey	ENSEASG00005019895	Skip	ENSEAST00005082992.1 OCA2-205<br>ENSEAST00005031948.2 OCA2-203	https://www.ensembl.org/Equus_asinus/Gene/Summary?db=core;g=ENSEASG00005019895;r=2:157758925-158119915

Table S2:
