# Supplementary material for: From paleness to albinism: Contribution of OCA2 exon 10 skipping to hypopigmentation
Source: PLoS Genet. 2025 Sep 25;21(9):e1011801. doi: 10.1371/journal.pgen.1011801 (PMC12463227; doi:10.1371/journal.pgen.1011801)
Supplement: S3 Table — (a) Association to skin colour. (b) Association to hair colour. (PDF) [file pgen.1011801.s011.pdf]

(a)

rs1800404	rs1800407	Haplotype frequencies Very Fair N = 31 800	Fair N = 291 293	Light Olive N = 71 886	Dark Olive N = 6 189	Brown N = 1 680	Black N = 23
T	C	0.7925	0.7923	0.7781	0.7721	0.7842	0.7608
T	T	0.0042	0.0029	0.0014	0.0014	0.0003	0
C	C	0.1130	0.1218	0.1401	0.1509	0.1450	0.1522
C	T	0.090	0.0831	0.0804	0.0756	0.0705	0.0870
**Additive haplotypic Odds Ratio**							
T	C	1.077 [1.049 - 1.106]	ref	0.853 [0.839 - 0.868]	0.787 [0.748 - 0.827]	0.831 [0.754 - 0.916]	0.765 [0.438 - 1.336]
T	T	1.569 [1.365 - 1.804]	ref	0.437 [0.374 - 0.511]	0.405 [0.243 - 0.674]	-	-
C	C	ref	ref	ref	ref	ref	ref
C	T	1.169 [1.126 - 1.212]	ref	0.840 [0.819 - 0.862]	0.734 [0.678 - 0.795]	0.716 [0.612 - 0.837]	0.821 [0.640 - 1.052]
**Under the Assumption of proportionnal Odds**							
T	C	0.853 [0.841 - 0.866]	$p = 2.05\ 10^{-101}$				
T	T	0.472 [0.426 - 0.523]	$p = 2.56\ 10^{-46}$				
C	C	ref					
C	T	0.813 [0.796 - 0.831]	$p = 1.10\ 10^{-78}$				

(b)

rs1800404	rs1800407	Haplotype frequencies Black N =16 679	Dark Brown N = 148 861	Light Brown N = 166 977	Blonde N = 46 666
T	C	0.76204	0.77222	0.79861	0.81809
T	T	0.00179	0.00228	0.00272	0.00385
C	C	0.12821	0.12735	0.12335	0.12305
C	T	0.10796	0.09815	0.07531	0.05502
**Additive haplotypic Odds Ratio**					
T	C	0.917 [0.886 - 0.949]	0.936 [0.922 - 0.95]	ref	1.027 [1.005 - 1.05]
T	T	0.641 [0.483 - 0.852]	0.807 [0.725 - 0.899]	ref	1.419 [1.244 - 1.619]
C	C	ref	ref	ref	ref
C	T	1.399 [1.333 - 1.468]	1.263 [1.235 - 1.291]	ref	0.732 [0.706 - 0.759]
**Under the Assumption of proportionnal Odds**					
T	C	0.932 [0.92 - 0.944]	$p = 7.24\ 10^{-27}$		
T	T	0.692 [0.633 - 0.756]	$p = 3.44\ 10^{-16}$		
C	C	ref			
C	T	1.376 [1.35 - 1.402]	$p = 7.99\ 10^{-236}$		

Table S3
