# Supplementary material for: From paleness to albinism: Contribution of OCA2 exon 10 skipping to hypopigmentation
Source: PLoS Genet. 2025 Sep 25;21(9):e1011801. doi: 10.1371/journal.pgen.1011801 (PMC12463227; doi:10.1371/journal.pgen.1011801)
Supplement: S4 Table — Allele frequencies provided in gnomAD v4.1.0 (including the UK Biobank). For each variant, the population with the highest frequency is highlighted in red, the one with the lowest frequency in green. NA: non applicable (too many citations). (PDF) [file pgen.1011801.s012.pdf]

Variant	rs code	Publications	Allele frequency	Number of homozygotes	Frequency						
					Europe	Africa	South Asia	East Asia	Middle East	Amixed americans	Rest of the world
p.Val350Met c.1048G>A	rs533478642	PMID10649493 R.Kerr and al. 2000<br>PMID37882226 Beili Jiang and al. 2024	9.79E-05	2	2.50E-05		1.30E-03	2.20E-05			1.40E-04
p.Leu354Pro c.1061C>T	rs1008522653	-	6.82E-06	0							
p.Ala355Ala c.1065G>A	rs1800404	na	7.17E-01	438294	8.10E-01	2.00E-01	3.90E-01	4.00E-01	6.70E-01	5.40E-01	6.80E-01
p.Ala355Val c.1064C>T	rs570914443	PMID23504663 Dimitre R Simeonov ans al. 2013<br>PMID28266639 Mohsin Shahzad ans al. 2017<br>PMID37882226 Beili Jiang and al. 2024	1.46E-04	4	1.20E-05	1.30E-05	2.20E-03	4.50E-05		6.70E-05	2.10E-04
p.Leu361Val c.1081C>G	rs769767739	PMID37882226 Beili Jiang and al. 2024	6.20E-06	0							
p.Ala368Val c.1103C>T	rs61745150	PMID28451379 Jackson Gao and al. 2017<br>PMID32830442 Daniel Jackson and al. 2020<br>PMID37882226 Beili Jiang and al. 2024	1.32E-04	1	2.50E-06	2.00E-02				5.00E-05	1.10E-04
p.Ile370Thr c.1109T>C	rs34731820	PMID10649493 R.Kerr and al. 2000<br>PMID32966289 Jenna E Rayner and al. 2020<br>PMID37882226 Beili Jiang and al. 2024	6.13E-04	9	5.00E-06	1.20E-02	5.00E-05			3.80E-04	7.20E-03
p.Gly371= c.1113C>T	rs1800405	PMID21541274 Markus N Preising and al. 2011<br>PMID37882226 Beili Jiang and al. 2024	1.40E-02	479	1.10E-02	3.00E-03	2.50E-02	1.60E-04	1.20E-02	9.80E-02	1.30E-02
p.Asp372Asn c.1114G>A	rs370353320	-	1.92E-05	1	6.80E-06	9.30E-05	1.50E-04				3.20E-05

Table S4
