# Supplementary material for: From paleness to albinism: Contribution of OCA2 exon 10 skipping to hypopigmentation
Source: PLoS Genet. 2025 Sep 25;21(9):e1011801. doi: 10.1371/journal.pgen.1011801 (PMC12463227; doi:10.1371/journal.pgen.1011801)
Supplement: Attachment — Submitted filename: Response to reviewers.pdf [file pgen.1011801.s014.pdf]

*Plos Genetics*
Editorial Office
Point by point answers to the Reviewers

Bordeaux, August the 25th, 2025

Dear editor, dear reviewers,

We have carefully addressed the points raised after reviewing the original version of the following article submitted to *Plos Genetics* July 2025: Contribution of *OCA2* exon 10 skipping to hypopigmentation" (PGENETICS-D-25-00764R1), and made the necessary modifications in the revised manuscript. We are sincerely grateful to both reviewers as we feel the revision process helped us to substantially improve the manuscript.

Here are our point-by-point answers (highlighted in blue) to each of the reviewers' concerns.

Reviewer #1: The authors of "From paleness to albinism: Contribution of OCA2 exon 10 skipping to hypopigmentation," present an interesting investigation of a collection of variants with a range of population frequency and their effect on alternative splicing of OCA2 exon 10 and pigmentation phenotype. I believe this work is important to the fields of population genetics, pigmentation biology, and clinical genetics. The breadth of this work is impressive, including variant analysis, functional assays of splicing, evolutionary alignments, phenotype association, and modeling molecular consequences on the protein level. I believe this is a worthy study, but I do suggest the following revisions:

Major issues:

1. Computational predictors of splice site disruption (i.e. SpliceAI and Pangolin) are low for the variants described in Table 1 and NM_000275.3:c.1080C>T, the previously described p.Ser360= variant. The authors should explore other biological explanation of these variants' obvious effect on exon 10 skipping. It would be worthwhile to explore whether these variants fall within eCLIP-seq peaks in data available through publicly available databases. No additional experiments are needed, but RNA-protein interaction with splicing factors may explain this phenomenon better than splice-site disruption and would be a worthwhile investigation.

SpliceAI and Pangolin are routinely used in the context of molecular diagnosis, reason why we provided the corresponding scores in Table 1.

In addition, we tested several *in silico* prediction tools for RNA-protein interaction modifications[1].

The main predictions were: c.1048G>A : Loss of ESE site SC35; c.1061T>C : Loss of ESE site SRp40; c. 1064C>T : Loss of ESE site SF2/ASF; c.1081C>G : Loss of ESE site SC35 Loss of ESS site hnRNPK; c.1109T>C : Addition of ESE sites SRp40 and SF2/ASF and ESS sites Fox-1 et Fox-2 ; c.1114G>A : no change; and for the pathogenic reference c.1080C>T: Loss of ESS hnRNPK, Addition of 2 ESS sites hnRNPI. Loss of 2 ESE sites SC35). None of the candidate RBPs were reported as targeting *OCA2* RNA

[1] ESEFinder (Cartegni L. et al., ESEfinder: a web resource to identify exonic splicing enhancers. Nucleic Acid Research, 2003, 31(13): 3568-3571.) and SpliceAid (http://www.introni.it/splicing.html Piva et al 2009, Bioinformatics PMID: 19261717).

in the eCLIP-seq database using the Postar3 platform[2] which lists a total of 3 RBPs for *OCA2* RNA: UTP3, EIF4G2 et AGGF1, none of which target the exon 10 region.

To follow the recommendations of the reviewer, we browsed the ENCODE project portal and queried the RNA-Protein interactions (ENCORE) datasets[3]. We selected eCLIP experiments for all 208 reported RNA binding protein targets and visualized eCLIP peaks through the UCSC genome browser in the region of *OCA2* exon10 and flanking regions. Only one peak meeting this criterion was detected at chr15:27990581-27990622, which corresponds to the second half of exon 10. This peak identifies WD repeat-containing protein 3 (WDR3). WDR3 is primarily characterized in the context of ribosomal RNA processing, thus rendering any assessment related to its putative functions in alternative splicing of the *OCA2* gene weak. Moreover, the peak was obtained after eCLIP experiments in K562 cells (bone marrow derived) only, which express very low levels of *OCA2* mRNA[4], questioning the relevance of this signal in the context of pigment cells.

Overall, we felt that these queries and the resulting predictions do not add relevant information to our study.

2. Along these same lines, NM_000275.3(OCA2):c.1045-9T>G, discussed in "Vulnerability of human OCA2 to exon 10 skipping relies on the combination of exonic and intronic sequences" and Figure 3c does have significant SpliceAI scores 0.24 (acceptor loss -9bp (the canonical acceptor site)) 0.22 (acceptor gain, +54bp), and Pangolin score (Splice Loss 0.47, -9bp). Does this variant cause exon 10 skipping or does it create an additional splicing product with partial retention of the 5' end of exon 10 (i.e. new splice site has now moved +45bp into exon 10), as the SpliceAI scores predict? Is there evidence or literature of exon 10 skipping vs. exon 10 missplicing for this variant?

All variants of exon10 that induced precise exon skipping in the minigene assay (as verified by sequencing) and for which we had patient samples (for instance c.1080C>T) show exact skipping in patient cells with the last nucleotide of exon 9 followed by the first nucleotide of exon 11 (checked by sequencing)[5]. When tested by minigene assay, c.1045-9T>G as well induces precise skipping of exon 10 in the unique 263 bp band as shown by the sequence below.

**c.1045-9T>G: one unique band 263 bp with a 100% skipped exon 10**

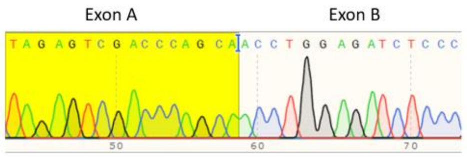


**Sequence of the RT-PCR product from minigene carrying c.1045-9T>G in *cis* to c.1065G>A in pSPL3b (details of construct in the materials and methods section and sup table)**

---

[2] the POSTAR3 platform: http://111.198.139.65/RBS.html

[3] The EncoRE project: https://www.encodeproject.org/encore-matrix/?type=Experiment&status=released&internal_tags=ENCORE

[4] As described in the Human Protein Atlas: https://www.proteinatlas.org/ENSG00000104044-OCA2/single+cell

[5] Michaud et al. Unsuspected consequences of synonymous and missense variants in OCA2 can be detected in blood cell RNA samples of patients with albinism. Pigment Cell Melanoma Res. 2024 Sep;37(5):534-545. doi: 10.1111/pcmr.13123. Epub 2023 Aug 31. PMID: 37650133. [our ref 13]

We have not had access to RNA samples of a patient carrying c.1045-9T>G, yet the minigene assay indicates 100% of exact skipping of exon 10, with no contamination by spliced or misspliced RT-PCR products. To our knowledge, no studies of the RNA profile resulting from this variant in patients has been reported in the literature.

Minor Issues

3. Lines 115-116: The authors state that the probands with the c. c.1048G>A/p.Val350Met and c.1081C>G variant are reported to have an additional deep intronic OCA2 variants in trans. Given the autosomal recessive mode of inheritance of OCA, I think it is an oversight not to describe these additional variants. Are there any computational predictors that suggest biological consequences (regulation, alternative splicing, conservation, CADD score) of these variants, that make it worth mentioning? If so, what are the deep intronic variants? Do the authors consider these variant to be *[missing word, supposedly: "pathogenic?"]*

We rephrased the description of the two intronic variants in *trans* to make it clear that they are both of unknown significance.

The details are as follow:

Variant c.1048G>A/p.Val350Met: in the Cypriot patient of our cohort, the only potentially pathogenic variant in *trans* is c.2080-11930G>T. This intronic variant is absent from ClinVar; predicted by RNA-Splicer to induce a pseudo-exon with a premature termination codon. No patient RNA sample was available at the time of writing to test this prediction. So, this deep intronic variant is still VUS. Note that c.1048G>A/p.Val350Met is reported in *trans* to c.1503+5G>A (ClinVar: RCV000193557/) [our ref 25].

Variant c.1081C>G/p.Leu361Val has been identified only once in one patient of our cohort. The only rare variant that we find in *trans* is intronic c.574-53C>G reported VUS in ClinVar (RCV004598362/). It is predicted by RNA-Splicer to activate a new acceptor site with a premature termination codon. Testing this prediction on patient RNA has not yet been achieved due to unavailability of patient sample.

4. Table 1: What were the specifications for the SpliceAI scores (i.e. masked vs. unmasked, max distance parameters) and what was the predicted effect (acceptor/donor loss/gain) , and delta-bp of the SpliceAI prediction? There are also minor errors with the values in Table 1, none of analytical significance: c.1114G>A SpliceAI: 0.07 (donor loss, -142bp), c.1061T>C Pangolin = 0.05 (Splice Gain); c.1081C>G Pangolin = 0.05 (Splice Gain). These may have been updated in the databases since the time of writing.

Table 1 has now been corrected providing the details for SpliceAI scores (unmasked, 500 bp max distance) with effect type (acceptor/donor loss/gain) and distance in bp, changing some scores that have been updated in the databases since the time of writing.

5. The allele counts of three of the variants (c.1048G>A, c.1064C>T, c.1109T>C) are notably high. These variants seem to be ancestry-specific as expounded upon briefly in the Discussion in lines 435-436 and Table S4, but I think this information is important enough to describe in the Results in discussion of Table 1.

A sentence was added to comment allelic frequencies and HMZ scores of these variants in the Result section under Table 1. We also rephrased the Discussion sentences dealing with the allele frequencies worldwide vs ancestry specific to make them more understandable.

6. Figure 1C: given that these are RT-PCR samples, these lanes should be labeled with label with genomic variant, not protein variant.

The figure labels have been corrected according to this comment.

7. There is a typo on line 297. I believe it is supposed to read "haplotype rs1800404-T/rs1800407-T" and not "haplotype rs1800404-T/rs1800404-T"

Thanks to the reviewer to have spotted this typo which is now corrected.

8. Lines 428-430: the authors recommend minigene assay for novel exon 10 mutations to test for splicing effect. While this may provide nominal research value, testing every exon 10 VUS via minigene is likely not feasible for many clinics or clinical geneticists in routine patient care. Confirming increased exon 10 skipping on a patient level would not alone be enough evidence for a promotion from VUS to Likely Pathogenic or Pathogenic via ACMG guidelines, and it is unclear how it would inform patient care without ASO treatments available.

We agree. This sentence was both irrelevant and useless and has therefore been removed from the text.

9. Methods: There is mention of a "local cohort" throughout the manuscript, yet the Materials and Methods section does not detail number of participants or inclusion/exclusion criteria. Assuming this is the same cohort from this group described in reference 12, it would be helpful to (very) briefly describe the cohort and make this overlap clearer in the text.

A paragraph describing the local cohort has now been added to the Materials and Methods.

10. There is a lack of consistency in variant descriptors. The authors switch from HGVS notation and rsID descriptors throughout the manuscript. Notably mentioned in lines 271-272: "c.1065G>A (referred to as rs1800404-T in population genetic studies and here after)," yet c.1065G>A and rs1800404-T are still used interchangeably throughout the manuscript

We apologize for the lack of consistency in using c. or rs identification of variants (c.1065G>A / rs1800404-T). We revised the manuscript according to this comment.

11. Finally, there are some casual or out of place word choices throughout the manuscript that should be revised. Two examples are in brackets.

a. "Pathogenic variants that cause albinism express themselves as a continuous spectrum from {extreme whiteness} and severe visual impairment to marginally subnormal vision and mild sensitivity of the skin to UV-light." (lines 53-55).

b. "All animal tissue samples used in this study were acquired from {left-over} post-mortem tissues from other studies involving wild-type control animals" (lines 504-505).

The whole manuscript has now been carefully revised by a native English speaker (co-author: Dr. David Hicks).

Reviewer #2: The authors present a series of functional experiments in transfected HeLa cells using minitemplates and chimeric constructs of human and mouse OCA2 exon 10 to demonstrate that the ratio of skipping of human OCA2 exon 10 occurs specifically in humans. This processing is modified by the rs1800404 SNP located within that exon. The data is quite convincing. The genetic association studies they conduct between skin and hair phenotypes and the rs1800404 (in exon 10) - rs1800407 (exon 13) haplotypes in the large human collection provided by the UK biobank is sufficient evidence to support the model presented in Figure 8.

Minor comments:

1. In the Introduction the authors state Tyrosinase catalyzes the reaction "…by converting tyrosine into L-Dopa". It is now understood that Tyrosinase converts tyrosine to dopaquinone (DQ), but that DOPA can assist in that conversion. See Ito and Wakamatsu 2008, Ito, S., & Wakamatsu, K. (2008). Chemistry of mixed melanogenesis--pivotal roles of dopaquinone. Photochem Photobiol, 84(3), 582-592. doi:10.1111/j.1751-1097.200
Our sentence was misleading with respect to the chemistry of the initial steps catalyzed by the Tyrosinase. We have now reformulated this sentence and quote a recent review by Wakamatsu et al.[6],

2. In the study of the rs1800404-rs1800407 haplotypes in the large collection provided by the UK biobank, is it possible to also do an association study with eye colour? Note that these SNPs were associated with blue eye colour in an earlier study, Table 3 of Duffy et al, Am J Hum Genet. 2007 Feb;80(2):241-52
A three-single-nucleotide polymorphism haplotype in intron 1 of OCA2 explains most human eye-color variation.

The association of SNPs or haplotypes with self-reported iris colour can be tested in the UK Biobank database. In this study, we restricted our analysis to skin and hair pigmentation as these phenotypes depend on fewer complex parameters (iris colour depends on combined involvement of iris stromal melanocytes and posterior pigmented epithelium) and more directly reflect the concept of hypopigmentation, particularly with regard to skin phototype. As discussed in our article, our future aim is to study the effect of rs1800404 in the eye, particularly in the retinal pigmented epithelium. This layer of cells plays a key role in retinogenesis, which explains most of the visual impairments experienced by patients with albinism. This study will also include an association analysis to measured (rather than self-reported) iris pigmentation.

3. In the conducting the analysis of the rs1800404-rs1800407 haplotypes in the UK Biobank cohort, did the authors consider if there was any effect of the major blue/brown eye colour associated SNP rs12913832 on those haplotypes? Is this possible to do rather than be extrapolated in the Discussion. What would be the relative contribution of rs12913832 vs rs1800404/rs1800407 to the penetrance of phenotypes?

In the discussion section of our work, we did not intend to extrapolate on the effect of rs12913832 + rs1800404/rs1800407 on pigmentation diversity in the general population. However, we deemed it relevant to mention rs12913832 in the context of molecular diagnosis for patients with albinism and suspected high levels of exon 10 skipping. Unlike in databases such as the UK Biobank, it is possible to access the diplotypes of some of the patients. It is then possible to try and predict the *cis*-modulating

---

[6] Wakamatsu, Zippin and Ito: Pigment Cell Melanoma Res. 2021 July ; 34(4): 730–747. doi:10.1111/pcmr.12970 [ref 9].

effect of rs12913832-C (loss of enhancer) or rs12913832-T (enhancer+) on a variant or haplotype that would significantly increase exon 10 skipping, taking into account the allelic combination in *trans*.

We did not try and investigate the relative contribution of rs12913832 versus rs1800404-rs1800407 to skin and hair phenotypes in the UK Biobank database. A rs12913832/rs1800407 haplotype analysis in healthy populations has previously been reported[7]. This work illustrates the challenging interpretation of unphased genotype association studies when each SNV acts at different levels of expression with combined *cis* and *trans* interactions. Kidd et al. concluded: "Even assuming the haplotypes affect phenotype additively, to estimate the three different fitness parameters associated with the three derived chromosomes seems beyond the power of the existing data. We leave such estimation to others." Adding a third SNV (here rs1800404) to such an analysis would raise the number of possible phased diplotypes from 10 to 36. While this is beyond the scope of our study, we agree that such an analysis would be valuable provided it is statistically relevant. This could explain why rs1800407 has a poor predictive value for skin and hair colour in European populations, and call into question its causal involvement[8].

4. Is it possible to demonstrate the level/ratio of OCA2 exon10 skipping based on genotypes by RT-PCR directly in RNA extracted from skin tissue or cultured clonal human melanocytic cells.

As we previously showed[9], invasive biopsies and time-consuming melanocyte-derived cell culture can be avoided as excessive *OCA2* exon 10 skipping can be detected by RT-PCR on RNA extracted from blood samples. Since this publication, this procedure is routinely applied in our diagnosis lab for patients with suspected splicing variant in *OCA2* or *SLC45A2* and available blood samples from the patient and his/her parents.

5. Can the two OCA2 protein isoforms (+/-exon10) be detected and quantitated on protein extracted from tissue or human cultured clonal melanocytic cells based on OCA2 genotype.

To our knowledge, the expression of the alternative isoform lacking a stretch of 24 amino acids (from p.349 to p.372) has not yet been confirmed by experimentation. Western blots using the available antibodies cannot discriminate between the two isoforms due to poor antibody performance and similar isoform sizes (Pr. Mickael Marks, expert in OCA2 protein functional studies, personal communication).

We searched for evidence of ΔE10-OCA2 by querying mass spectrometry databases (both public and personal) for specific peptides containing contiguous amino acids p.348Glu (encoded by the last codon of exon 9) and p.373Arg (encoded by the first codon of exon 11).

**Public database**: No compatible peptides were detected in any of the following datasets.

Proteomics Identification Database PRIDE https://www.ebi.ac.uk/pride/
SRmAtlas https://srmatlas.org/#

---

[7] Kidd KK, et al. The distinctive geographic patterns of common pigmentation variants at the *OCA2* gene. Sci Rep. 22 sept 2020;10(1):15433), [our ref 38]
[8] Navarro-López B et al. Exploring Eye, Hair, and Skin Pigmentation in a Spanish Population: Insights from Hirisplex-S Predictions. Genes. 2024 Oct 16;15(10):1330. doi: 10.3390/genes15101330. PMID: 39457454; PMCID: PMC11507238
[9] Michaud et al. Unsuspected consequences of synonymous and missense variants in OCA2 can be detected in blood cell RNA samples of patients with albinism. Pigment Cell Melanoma Res. 2024 Sep;37(5):534-545. doi: 10.1111/pcmr.13123. Epub 2023 Aug 31. PMID: 37650133. [our ref 13],

MassIVE https://massive.ucsd.edu/ProteoSAFe/static/massive.jsp
Panorama Targeted Proteomics Knowledge Base https://panoramaweb.org/home/project-begin.view
Peptide Atlas https://peptideatlas.org/

Only one call with a low confidence score was identified in the Generalized Proteomics data Meta-analysis database **FERPSLTHV**, see link[10]. This peptide maps the junction encoded by exon9 and 11 (highlighted in blue on the protein sequence below, exon 10 encoded-region in green)

The confidence scores for this peptide are however too low to support the identification of ΔE10-OCA2.

**Personal data**: Among the six peptides (highlighted in red on the protein sequence) that were recovered from cultured melanocytes that express the full length and skipped transcript, none do map the region of interest.

MHLEGRDGRRYPGAPAVELLQTSVPSGLAELVAGKRRLPRGAGGADPSHSCPRGAAGQSSWAPAGQEFASFLT
KGRSHSSLPQMSSSRSKDSCFTENTPLLRNSLQEKGSRCIPVYHPEFITAEESWEDSSADWERRYLLSREVSGLSAS
ASSEKGDLLDSPHIRLRLSKLRRCVQWLKVMGLFAFVVLCSILFSLYPDQGKLWQLLALSPLENYSVNLSSHVDSTL
LQVDLAGALVASGPSRPGREEHIVVELTQADALGSRWRRPQQVTHNWTVYLNPRRSEHSVMSRTFEVLTRETV
SISIRASLQQTQAVPLLMAHQYLRGSVETQVTIATAILAGVYALIIFE**IVHRTLAAMLGSLAALAALAVIGD**RPSLTH
VVEWIDFETLALLFGMMILVAIFSETGFFDYCAVKAYRLSRGRVWAMIIMLCLIAAVLSAFLDNVTTMLLFTPVTIR
LCEVLNLDPRQVLIAEVIFTNIGGAATAIGDPPNVIIVSNQELRKMGLDFAGFTAHMFIGICLVLLVCFPLLRLLYWN
RKLYNKEPSEIVELKHEIHVWRLTAQRISPASREETAVRRLLLGKVLALEHLLARRLHTFHRQISQEDKNWETNIQEL
QKKHRISDGILLAKCLTVLGFVIFMFFLNSFVPGIHLDLGWIAILGAIWLLILADIHDFEIILHRVEWATLLFFAALFVL
MEALAHLHLIEYVGEQTALLIKMVPEEQRLIAAIVLVVWVSALASSLIDNIPFTATMIPVLLNLSHDPEVGLPAPPL
MYALAFGACLGGNGTLIGASANVVCAGIAEQHGYGFSFMEFFRLGFPMMVVSCTVGMCYLLVAHVVVGWN

Unfortunately, we do not have access to cells derived from the rare patients that are homozygotes for a full skipping inducing variant (such as c.1045-9T>G), in which case we would be able to test the expression of ΔE10-OCA2 by Western blot or mass spectrometry. Further investigations on the expression/function of ΔE10-OCA2 will therefore likely require recombinant protein engineering.

---

Respectfully,

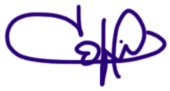

Sophie JAVERZAT, Univ. Bordeaux, INSERM U1211, France

[10] https://gpmdb.thegpm.org/~/protein/npep=0&homolog=69914&uid=69914&path=/gpm/archive/066/GPM06610107683.xml&label=ENSP00000261276&proex=-1
